# Phone and Web-Based Tobacco Cessation Treatment: Real-World Utilization Patterns and Outcomes for 11,000 Tobacco Users

**DOI:** 10.2196/jmir.999

**Published:** 2008-11-14

**Authors:** Susan M Zbikowski, Jenny Hapgood, Sara Smucker Barnwell, Tim McAfee

**Affiliations:** ^1^Clinical and Behavioral SciencesFree & ClearIncSeattleWAUSA

**Keywords:** Tobacco cessation, Internet, telephone, smoking

## Abstract

**Background:**

Phone-based tobacco cessation programs have been proven effective and widely adopted. Web-based solutions exist; however, the evidence base is not yet well established. Many cessation treatments are commercially available, but few integrate the phone and Web for delivery and no published studies exist for integrated programs.

**Objective:**

This paper describes a comprehensive integrated phone/Web tobacco cessation program and the characteristics, experience, and outcomes of smokers enrolled in this program from a real-world evaluation.

**Methods:**

We tracked program utilization (calls completed, Web log-ins), quit status, satisfaction, and demographics of 11,143 participants who enrolled in the Free & Clear Quit For Life Program between May 2006 and October 2007. All participants received up to five proactive phone counseling sessions with Quit Coaches, unlimited access to an interactive website, up to 20 tailored emails, printed Quit Guides, and cessation medication information. The program was designed to encourage use of all program components rather than asking participants to choose which components they wanted to use while quitting.

**Results:**

We found that participants tended to use phone services more than Web services. On average, participants completed 2-2.5 counseling calls and logged in to the online program 1-2 times. Women were more adherent to the overall program; women utilized Web and phone services significantly (*P* = .003) more than men. Older smokers (> 26 years) and moderate smokers (15-20 cigarettes/day) utilized services more (*P* < .001) than younger (< 26 years) and light or heavy smokers. Satisfaction with services was high (92% to 95%) and varied somewhat with Web utilization. Thirty-day quit rates at the 6-month follow-up were 41% using responder analysis and 21% using intent-to-treat analysis. Web utilization was significantly associated with increased call completion and tobacco abstinence rates at the 6-month follow-up evaluation.

**Conclusions:**

This paper expands our understanding of a real-world treatment program combining two mediums, phone and Web. Greater adherence to the program, as defined by using both the phone and Web components, is associated with higher quit rates. This study has implications for reaching and treating tobacco users with an integrated phone/Web program and offers evidence regarding the effectiveness of integrated cessation programs.

## Introduction

Telephone and Web-based cessation programs are widely available and used. Each year, approximately 1.1% to 1.7% of adult smokers receive tobacco cessation services via state quitlines across the United States [1] and many more receive services through their health plans and/or employers. Phone-based counseling has many benefits and has proliferated in availability since the 1990s. Currently, quitlines provide services in all of North America as well as many other countries across the world (including China, Taiwan, Hong Kong, Singapore, Thailand, South Korea, Australia, New Zealand, Brazil, and most countries in the European Union). Services range from mailed materials, referral to community resources, reactive and/or proactive counseling, medication information, and, in some cases, subsidized or free medication [2].

There is a large, high-quality evidence base for phone-based cessation counseling. Effectiveness has been established in dozens of large randomized trials and summarized in three meta-analyses published during the past decade [3-5]. The Cochrane Collaboration [5] published a systematic review that concluded that proactive phone counseling helps smokers who are trying to quit, improving quit rates by over 50%. Ossip-Klein and McIntosh offer a comprehensive review of this literature [1].

According to the updated Online Health Search Report [6] in 2006, 9% of Internet users searched for information on how to quit smoking. While vast quantities of information on tobacco cessation are available online, not all online information is designed for treatment [7]. In 2002, one in five websites related to cessation provided treatment information and only one-third of those that focused on treatment provided minimal coverage of the recommended components of the Public Health Services (PHS) Clinical Practice Guideline [3].

Several real-world evaluations of QuitNet, one of the most widely used online cessation services in the United States, have been conducted. Quit rates (7-day point prevalence rates) range from 7% to 30% at 3 months (intent-to-treat [ITT] and responder rates, respectively) among a population of general users (N = 1501) [8], to 13.2% to 17% at 6 months (ITT and responder rates, respectively) among a state population (Minnesota; N = 607) [9], to 12.8% to 42.9% at 12 months (ITT and responder rates, respectively) among an employee population (N = 1776) [10]. Furthermore, two of these evaluations found better outcomes associated with greater website use [8,10].

There are minimal data from randomized trials examining online cessation services. To date, results from four trials have been published. Three randomized trials have demonstrated effectiveness of online cessation approaches [11-13], and one study found no improved outcomes above individual counseling [14]. The quit rates from these studies are fairly comparable to those from the real-world evaluations reported above; however, the follow-up periods were often shorter (eg, 6 and 11 weeks).

In the one study [14] incorporating an online intervention with standard treatment that included three in-person 20-minute counseling sessions and medication (bupropion SR), there were no differences in outcomes (7-day point prevalence) between groups at 3 or 6 months. However, among those participants randomized to the online intervention, average log-ins per week and abstinence status were significantly related at 3 and 6 months (odds ratio [OR] ranged from 1.6-1.8). That is, greater use of the online intervention per week was associated with greater quit outcomes.

To date, there are no studies that describe the efficacy or effectiveness of integrated tobacco cessation treatment including telephone counseling and online services. The purpose of the current study is to present results of an evaluation of the Quit For Life integrated phone/Web program, which is widely available through state quitlines, health plans, and employers across the United States. The Free & Clear Quit For Life phone program has been commercially available for nearly 20 years. In 2006, an interactive online program was integrated into the standard program. The integration of phone and Web modalities is a critical aspect of this novel program. The approach is that all participants are provided with a comprehensive program that includes counseling calls, online services, and printed materials. Participants do not choose between these services but receive all of them over the course of the program.

The potential advantage of an integrated phone/Web program is that it offers individualized one-on-one counseling with a cessation expert in addition to the dynamic online support that can be available at any time. While phone-only and Web-only programs have their advantages, a combined program has the potential to improve outcomes and efficiency by providing tools and integrated services that appeal to a wide variety of tobacco users with different needs and learning styles. In this paper we describe (1) the characteristics of the participants using the program, (2) the phone and Web utilization rates, and (3) quit outcomes and satisfaction.

## Methods

This study examined the experience of 11,143 enrollees in the Free & Clear Quit For Life Program, a smoking cessation program including proactive phone-based counseling, an interactive website, and printed Quit Guides. To be eligible for this study, a participant had to be a tobacco user who spoke English, be 18 years or older, be enrolled in the program through his or her health plan or employer between May 2006 and October 2007, have access to an email account, and consent to follow-up at 6 months. State participants were excluded due to lack of systematic follow-up.

### Description of the Intervention

The Free & Clear Quit For Life phone program has been commercially available for nearly 20 years. The program is grounded in social cognitive theory [15,16] and incorporates the strategies for effective tobacco dependence treatment outlined in the US PHS Clinical Practice Guideline. The effectiveness of the program has been demonstrated in three randomized trials [17-19] and in several real-world evaluations (published evaluations include [20-22]). From its inception, the Quit For Life Program has included individualized telephone counseling sessions and printed Quit Guides. In 2006, the interactive online program (Web Coach) designed to complement the phone-based treatment sessions was added.

The Quit For Life Program is available to participants through their employer or health plan. Participants enroll in the program directly by phone or online. Once registered, participants receive up to five one-on-one proactive phone counseling sessions, access to the interactive website (Web Coach), and printed self-help materials (Quit Guides). Phone counseling sessions are with an intensively trained tobacco treatment specialist (Quit Coach). The counseling calls are designed to provide practical expert support to help participants develop problem-solving and coping skills, secure social support, and design a plan for successful cessation and long-term abstinence. Calls are scheduled at times convenient for the caller and at relapse-sensitive intervals. Participants can also call a 1-800 number as many times as they want for additional support between calls. For all proactive counseling calls, we make up to three attempts to reach a participant for each of the ongoing, proactive calls. The number of calls completed may vary due participants declining a call or not being available to complete all calls.

**Figure 1:**
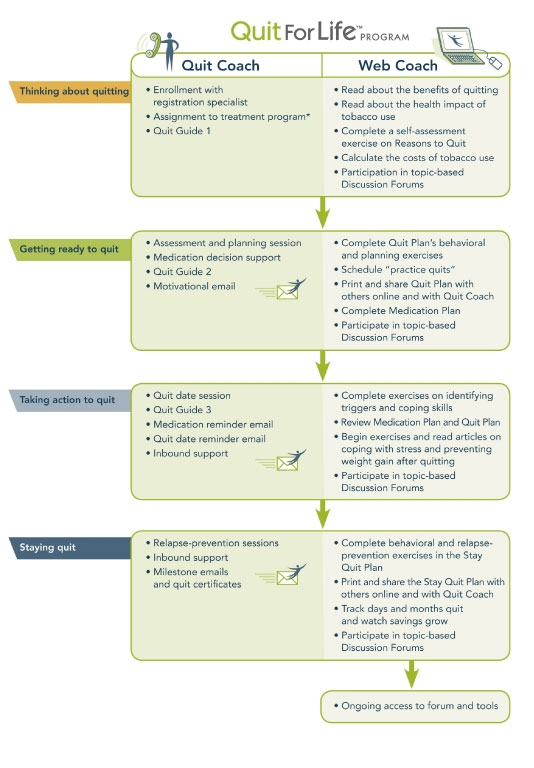
Interaction between the participant, Quit Coach, and Web Coach

During each call, the Quit Coach encourages participants to use the Web program and Quit Guides to document their quit plan and track their progress. Quit Coaches have real-time access to information that participants enter on the website. This information, along with information gathered during calls, is used by Quit Coaches to frame the focus of the counseling call. The interaction between the participant, Quit Coach, and Web program is shown in Figure 1.

**Figure 2:**
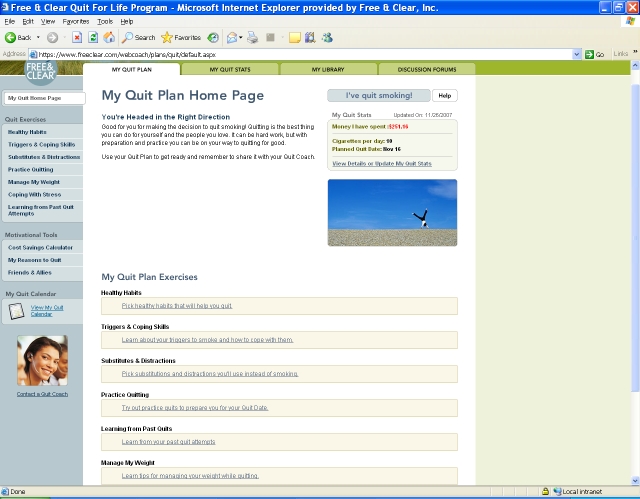
Quit plan

**Figure 3:**
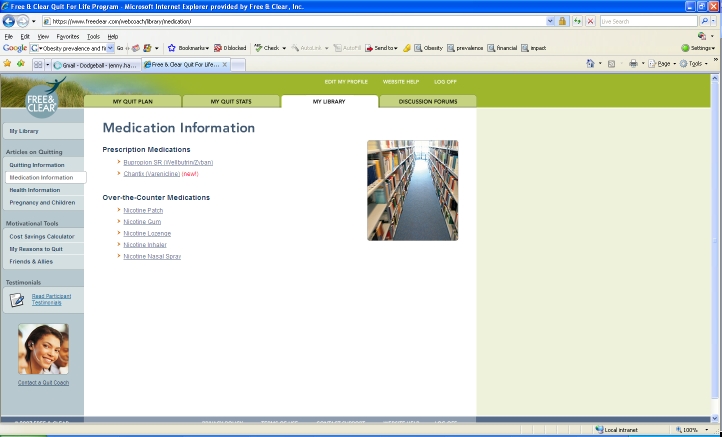
Library

**Figure 4:**
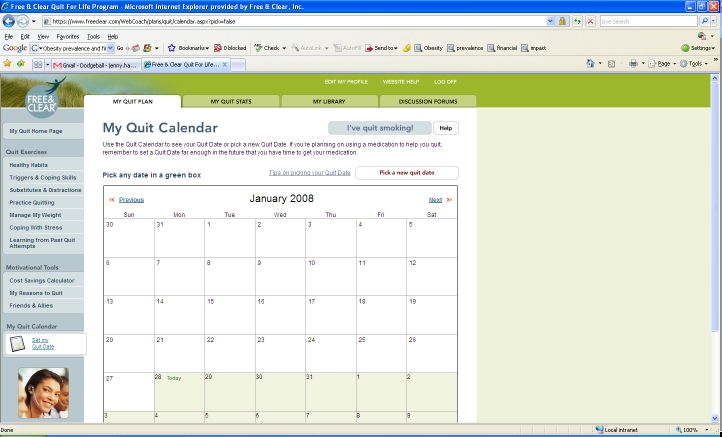
Quit calendar

**Figure 5:**
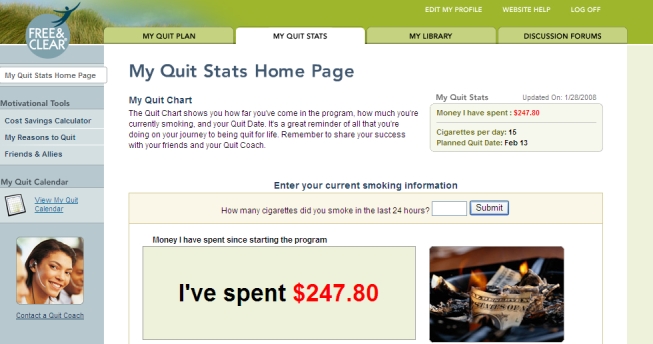
Progress tracker and cost calculator

**Figure 6:**
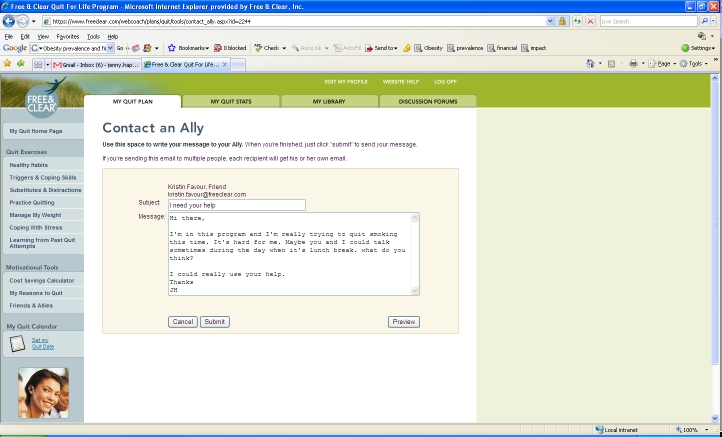
Email an ally

**Figure 7:**
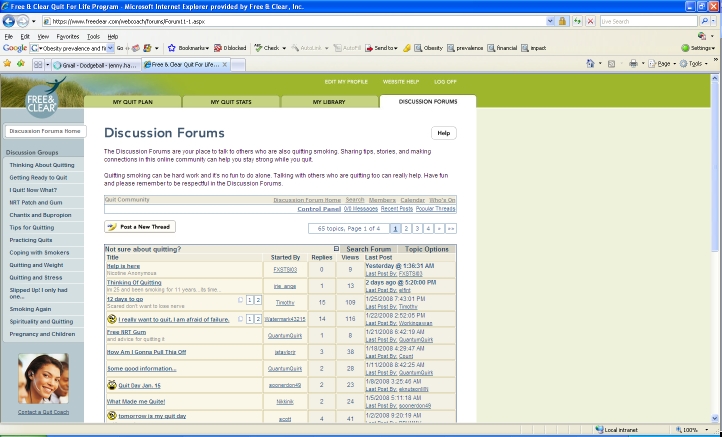
Discussion forum

**Figure 8:**
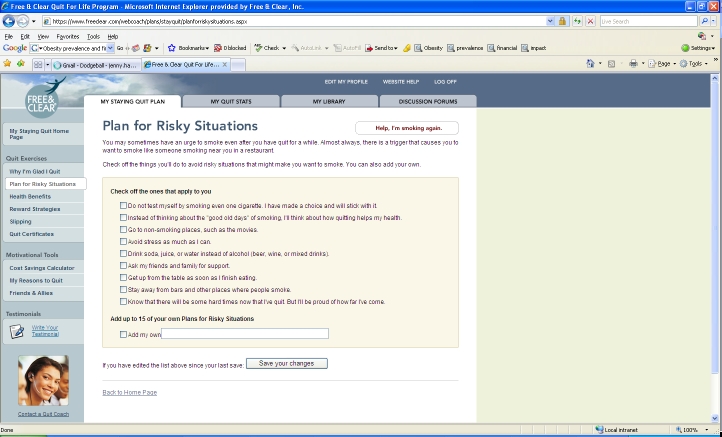
Relapse-prevention phase

The online component of the program (Web Coach) contains interactive tools and tailored content based on participants’ readiness to quit. As described in Table 1, key features of Web Coach include an interactive quit plan (Figure 2), educational content in an online library (Figure 3), quit calendar (Figure 4), cost calculator and progress tracker (Figure 5), tool to email friends, family, and other participants for support (Figure 6), and active discussion forums to interact with other members and the Quit Coaches (Figure 7). Participants can use the Web tools to gain greater awareness of their tobacco triggers, learn from past quit attempts, and develop their plan to cope with cravings, stress, and triggers. Participants build social networks with other smokers and ex-smokers enrolled in the program through the discussion forums and messaging functions available on the website. Quit Coaches moderate the forums and provide feedback to participants on a daily basis. Once a participant reports quitting tobacco, the website changes in its look, feel, and content to reflect that the participant has now quit and is actively working to prevent relapse (see Figure 8). This “Staying Quit” phase includes exercises and educational content for relapse prevention. When participants achieve quitting milestones (having quit for 1 month, 6 months, and 12 months), they are sent e-certificates recognizing their achievement.

**Table 1:** Key Web Coach features

Feature	Description
My Quit Plan	Includes tailored activities to help the participant discover his or her triggers to smoke, pinpoint effective coping strategies, and build a personalized quit plan. The plan is shared with the Quit Coach. Planning activities and content are tailored to the participant’s readiness to quit.
My Library	Contains educational articles on a variety of cessation-related topics including nicotine replacement therapy, over-the-counter cessation medications, tobacco use and chronic diseases, pregnancy and tobacco use, and weight gain and stress management and tobacco use.
My Quit Calendar	An interactive calendar that links to the participant’s overall program schedule and helps track the quit date.
My Discussion Forums	Discussion board with specific topics of interest related to cessation. Each group has hundreds of subtopics and thousands of postings from participants. Quit Coaches moderate the forums and post responses to participants’ questions.
My Quit Stats	Progress graph that displays the amount of money spent based on cigarettes consumed per day as well as money saved as participant decreases consumption.
My Reasons to Quit	Interactive exercise for participant to identify his or her reasons to quit, commit those to the Quit Plan, and share them with the Quit Coach.
Friends and Allies	Built-in email capability so participants may email friends, family, and coworkers who support them in their quit attempt.

### Measures

Participant demographics (age, gender), contract type (health plan, employer), current tobacco use (tobacco type, amount used), nicotine dependence (measured as time to first use upon waking), and readiness to quit were derived from information collected at registration and the participants first call with a Quit Coach. Phone counseling (number of live calls completed) and Web use (log-ins, forum visits) were tracked and recorded. Participants who consented to follow-up were contacted 6 months after enrollment in the Quit For Life Program and were administered a telephone survey. Those individuals who could not be reached via telephone after 11 attempts or did not have a working telephone number were sent a mailed survey. Approximately half of the participants responded to the survey (50.9%, N = 5675). The 10-minute follow-up survey addressed a variety of topics related to the participant’s experience with the Quit For Life Program, including program satisfaction and tobacco use behaviors/ abstinence. Abstinence data were obtained through self-report; no biochemical verification took place. Tobacco abstinence was defined as no tobacco use whatsoever in the previous 30 days. Participants were asked to rate their satisfaction with the program using a 4-point scale (very satisfied, somewhat satisfied, somewhat dissatisfied, very dissatisfied). Satisfaction was defined as indicating that one was “somewhat” to “very satisfied” with the Quit For Life Program services. The 30-day abstinence and satisfaction measures have been used in other published studies [19].

### Statistical Analysis

The results below summarize participant demographics and program utilization.

We conducted mean comparisons, chi-square analyses, and analyses of variance (ANOVA) to assess differences in counseling calls completed, Web Coach log-ins, and discussion forum log-ins by gender, age (18-25, 26-40, 41-60, 61+), contract type (health plan, employer), nicotine dependence (first cigarette of day within 5 minutes, > 5 minutes), cigarettes per day (< 15, 15-20, 21+), and readiness to quit (within 30 days, > 30 days). Within ANOVA, Scheffe’s post hoc comparisons identified specific between-group differences. Within contingency table variables with more than two groups, post hoc Bonferroni chi-square comparisons examined all between-group differences.

We stratified Web Coach use into three discrete categories (0 log-ins, 1-4 log-ins, 5+ log-ins) and conducted ANOVA to examine rates of Web use and average calls completed, satisfaction, and quit rates by level of Web use. We also classified counseling use into four discrete categories (0 calls, 1-2 calls, 3-4 calls, 5+ calls) and examined quit rates by counseling calls completed and Web log-ins (0 vs 1+ log-ins). The call level categories were derived from historical data that demonstrate different quit rates associated with these call completion levels. Since historical data were not available for Web use, we calculated three levels: no use (0 log-ins), low use (1-4 log-ins), and high use (5+ log-ins). Similar cutoff points have been used in other online studies [10,14].

Quit outcomes were examined using ITT analysis and responder analysis. In the ITT analysis, all participants eligible for follow-up were included in the analysis (N = 11,143), and nonresponders to the follow-up survey were assumed to be continued tobacco users. In the responder analysis, results were based on those who responded to the follow-up survey and provided outcome information (N = 5675, 50.9% of the sample), and no assumptions were made about the tobacco status of participants lost to follow-up.

Examination of the data identified several instances of very high Web utilization and discussion forum use among a small group of participants (eg, 266 Web Coach log-ins, 566 forum log-ins). It is notable that the distribution of program utilization data (ie, number of counseling calls, Web log-ins, forum log-ins) was highly skewed. We employed a strategy of two-sided, 1% trimming, removing .05% of all values (N = 117) from both sides of these two variable’s distributions to improve the estimate of central tendency. Trimming is a well supported method for robust examination of central tendency [23].

## Results

### Participants

Participants tended to be middle aged (mean = 43.0 years, SD = 10.8) and were evenly distributed among genders (54% female, 46% male); 83% of study participants enrolled in the program through their employer, while the remaining 17% enrolled through their health insurance plan. On average, participants smoked 12.5 cigarettes daily (SD = 12.4). Almost all (91.7%) participants reported planning to stop smoking within 30 days of their first contact with the program.

Overall, participants were less dependent on smoking than the general population of smokers. Responses to the single-item addiction index derived from the Fagerström Test for Nicotine Dependence [24] indicated that approximately one-third (35.6%) of respondents demonstrated a strong nicotine addiction (ie, smoking within 5 minutes of waking). Only 16% smoked more than a pack per day.

### Program Utilization and Adherence

Participants used phone counseling more than online services. Participants completed an average of two counseling calls (mean = 2.1, SD = 1.6, median = 2.0, interquartile range [IQR] = 2.0), utilized Web Coach an average of one time (mean = 1.1, SD = 1.9, median = 0.0, IQR = 1.0), and used discussion forums less often (mean = 0.51, SD = 2.1, median = 0.0, IQR = 0.0) than they logged in to Web Coach. However, utilization rates were much higher for those participants who engaged in phone or Web services at least once. Among those participants who took at least one telephone counseling call (N = 9376), the mean number of counseling calls was 2.5 (SD = 1.4, median = 2.0, IQR = 2.0). Among those participants who logged in to the Web services at least once (N = 5413), the mean number of Web Coach log-ins was 2.2 (SD = 2.2, median = 1.1, IQR = 1.0), and the mean number of discussion forum log-ins was 4.4 (SD = 4.6, median = 3.0, IQR = 3.0).

We stratified use of Web Coach into three discrete categories: 0 log-ins (50.8%), 1-4 log-ins (43.9%), and 5 or more log-ins (5.4%). ANOVA was then used to compare counseling calls completed among the three log-in groups. For the purpose of analysis, we also stratified number of calls completed into comparison groups: 0 calls (15.9%), 1-2 calls (47.8%), 3-4 calls (28.2%), and 5 or more calls (8.2%).

Demographic differences emerged among callers in terms of Web Coach and online discussion forum use, as well as number of calls completed in the cessation program. Women were significantly more likely than men to utilize online discussion forums and complete a greater number of calls. Furthermore, older callers were significantly more likely to complete more calls than younger callers. Younger callers (eg, 18-25 years) logged in to Web Coach significantly less often than middle-aged callers (ie, 41-60 years). Moderate smokers logged in to the Web Coach significantly more often than light or heavy smokers and logged in to discussion forums more often than light smokers. Callers eligible for tobacco cessation treatment through their employer were more likely to log in to Web Coach than those eligible for treatment through their health insurance plan (see Table 2).

**Table 2:** Web Coach and calls completed, by characteristic (N = 11,143). Numbers cited in statistics vary due to missing data for some analyses.

	No.	%	Calls, Mean (SD)	Web Coach Log-Ins, Mean (SD)	Forum Log-Ins, Mean (SD)
**Gender**					
Male	5170	46.4	2.1 (1.6)	1.1 (1.8)	.45 (1.9)
Female	5937	53.6	2.1 (1.6)	1.1 (2.0)	.57 (2.2)
Statistic			*t*_11,141_ = 3.0, *P=* .003	*t*_10,954_ = −2.0, *P=* .05	*t*_10,989_ = −3.0, *P=* .003
**Age Group**					
18-25	708	6.4	1.6 (1.4)	.83 (1.5)	.34 (1.3)
26-40	3670	32.9	1.8 (1.4)	1.0 (1.8)	.54 (2.1)
41-60	6346	57.0	2.3 (1.6)	1.1 (1.9)	.52 (2.2)
61+	419	3.8	2.8 (1.6)	1.1 (2.0)	.38 (1.9)
Statistic			F_3, 11,139_ = 146.0, *P<* .001	F_3, 10,952_ = 7.0, *P<* .001	F_3, 10,987_ = 2.4, *P=* .07
**Nicotine Dependence**					
First cigarette of day within 5 min of waking	2950	35.6	2.6 (1.4)	1.2 (2.0)	.59 (2.3)
First cigarette of day > 5 min of waking	5348	64.4	2.5 (1.4)	1.2 (2.0)	.56 (2.3)
Statistic			*t*_8296_ = 1.8, *P=* .07	*t*_8145_ = −1.1, *P=* .29	*t*_8171_ = .55, *P =* .58
**Cigarettes/Day**					
< 15	4996	53.4	1.6 (1.5)	1.0 (1.7)	.45 (1.9)
15-20	2846	30.4	2.6 (1.4)	1.2 (2.1)	.61 (2.4)
21+	1513	16.2	2.5 (1.4)	1.0 (1.9)	.50 (2.0)
Statistic			F_2, 9352_ = 505.4, *P<* .001	F_2, 9293_ = 10.4, *P* < .001	F_2, 9224_ = 5.6, *P* = .004
**Contract Type**					
Health Plan	1850	16.9	2.3 (1.6)	.96 (1.8)	.45 (2.0)
Employer	9105	83.1	2.1 (1.6)	1.1 (1.9)	.52 (2.1)
Statistic			*t*_10,953_ = 1.0, *P<* .001	*t*_10,768_ = −3.1, *P* = .002	*t*_10,804_ = −1.3, *P* = .20
**Readiness to Quit**					
Within 30 days	10,089	91.7	2.1 (1.6)	1.1 (1.9)	.51 (2.1)
> 30 days	914	8.3	1.9 (1.6)	1.1 (1.9)	.48 (2.1)
Statistic			*t*_11,001_ = 4.9, *P* < .001	*t*_10,816_ = −.17,*P*= .86	*t*_10,849_ = .46, *P* = .65

### Relation Between Web Coach Use and Call Completion

Respondents who logged in to Web Coach more frequently were significantly more likely to participate in greater numbers of counseling calls. All groups differed significantly. In fact, the small number of participants (5%) who logged on to Web Coach five or more times completed on average one more counseling call than other Web Coach users. Post hoc tests confirmed that all groups differed significantly (see Table 3).

**Table 3:** Web utilization and average calls completed, by Web Coach log-in group (N = 11,143)

Log-In Group	No. (%)	Average Live Calls, Mean (SD)	Statistic
0	5656 (50.8)	2.0 (1.6)	F_2, 11,140_ = 154.6, *P* < .001
1-4	4888 (43.9)	2.1 (1.5)	
5+	599 (5.4)	3.1 (1.7)	

### Program Outcomes

To assess outcomes, we examined rates of satisfaction and abstinence from tobacco for at least 30 days or more at 6 months after callers registered in the program. Among survey respondents, 92.1% were satisfied and 41.1% abstained from tobacco use for 30 days or more. When examining quit rates using the ITT analysis, the 30-day quit rate was lower (20.5%) due to attrition at follow-up.

Survey respondents who logged in to Web Coach five or more times were significantly more likely to be satisfied with their experience compared to those who never logged in. No other groups differed in terms of satisfaction. Survey respondents who logged in to the Web Coach more often were significantly more likely to have been abstinent from tobacco for 30 days or more. Even when using ITT analysis, individuals logging in to Web Coach more often were significantly more likely to cease tobacco use. In both responder and ITT analyses, all groups differed significantly (see Table 4). In both the responder and ITT analyses, a pattern emerged in which individuals who completed greater numbers of telephone calls and utilized Web Coach at least once reported significantly higher tobacco cessation rates overall (see Table 5).

Furthermore, a multivariate logistic regression revealed that both calls completed (OR = 1.56) and Web log-ins (OR = 1.14) were significant predictors of quit outcomes (30-day ITT) when controlling for age, gender, and cigarettes per day (*χ* ^2^_2_ = 953.7, N = 11,143, *P* < .001). Specifically, for each additional call, the odds of quitting increased by 56%, whereas for each additional log-in, the odds of quitting increased by 14%.

**Table 4:** Satisfaction and 30-day quit rates, by Web Coach log-in group

Log-In Group	Responders Satisfied, No. (%)	Responder 30-Day Quit, No. (%)	ITT 30-Day Quit, No. (%)
0	2268 (91.7)	873 (31.8)	873 (15.4)
1-4	2102 (92)	1156 (47.5)	1156 (23.6)
5+	353 (95.4)	253 (66.9)	235 (42.2)
Statistic	*χ*^2^_2_ = 6.1 N = 5128 *P=* .047	*χ*^2^_2_ = 242.6 N = 5551 *P* < .001	*χ*^2^_2_ = 292.7 N = 11,143 *P<* .001

**Table 5:** Quit rates (responder and ITT), by call level and dichotomous log-in level

	0 calls, No. (%)	1-2 calls, No. (%)	3-4 calls, No. (%)	5+ calls, No. (%)
**Responder Quit Rates^a^**				
0 log-ins	44/270 (16)	348/1179 (30)	345/ 960 (36)	136/332 (41)
1+ log-ins	51/192 (27)	458/ 1057 (43)	638/1156 (55)	262/405 (65)
**ITT Quit Rates^b^**				
0 log-ins	44/1047 (4)	348/2766 (13)	345/1427 (24)	136/416 (33)
1+ log-ins	51/720 (7)	458/2561 (18)	638/1711 (37)	262/495 (53)

^a^ *χ* ^2^_7_ = 351.4, N = 5551, *P* < .001. Chi-square compares quit vs not quit among each cell call group × Web log-in cell.

^b^ *χ* ^2^_7_ = 1045.8, N = 11,143, *P* *<* .001.

## Discussion

### Principal Results and Comparisons With Prior Work

To our knowledge, this is the first paper to describe the utilization and outcomes of an integrated tobacco cessation phone/Web program. Quit rates were 41% among survey responders and 21% when using the more conservative ITT analysis method. Quit rates were similar to other studies involving proactive phone counseling (see meta-analysis [4]) and higher than studies involving the Web alone [8-11]. Quit rates also were higher for those participants who had more log-ins and who completed more counseling calls. Similar results have been demonstrated in other studies. For example, Saul et al [9], Japuntich et al [14], and Graham et al [10] found that online use, specifically number of log-ins, was positively correlated with quit outcomes. Similarly, several studies on phone-based interventions have demonstrated a dose response [5,19,20,25].

This study also extends our understanding of the use of an integrated phone/Web program. Participants completed an average of 2-2.5 counseling calls and logged in to the online program an average of 1-2 times. We found that participants tended to use phone services more than Web services. In addition, we observed that nearly half of participants never logged in to the Web Coach. The lower log-in rates for the Web program are most likely due to the automated assignment of Web access to every participant regardless of his or her interest in using the Web program. Thus, participants who chose to log in after receiving automatic access to the Web program may represent a self-selection bias toward the Internet, while nonuse may indicate a bias toward phone counseling.

In a study of a worksite program with incentives, Graham et al [10] found that of the 28.5% of employees who chose to use a Web program over other cessation materials, less than 1% never returned to the website after registering. This suggests that people who deliberately choose a Web program as their cessation method are likely to use the Web program. We believe the higher levels of never logging in for this real-world integrated program are likely due to the passive nature of the Web enrollment process in our program, the availability of other services (mailed materials and phone counseling) to help the participants with quitting, as well as how the program was promoted by health plans and employers.

Even participants who never logged in took an average of two calls. However, participants who logged in more frequently also were more engaged in counseling by phone. Specifically, the small sample of participants who logged in five or more times took, on average, one call more than those participants who logged in fewer times. Thus, there appears to be a population of smokers trying to quit who utilize more services. Furthermore, we observed a trend for higher quit rates among participants who logged in to Web Coach at least once and took more calls. Given that this is not a randomized trial, we are unable to draw causal inferences.

There were several demographic differences in utilization. Women were more likely than men to use the discussion forums and phone counseling, suggesting that women may use these services to obtain social support while quitting. Older smokers completed more calls and used the Web program more often than younger smokers. In other evaluations that we have carried out, we found that younger smokers take fewer calls. We were disappointed to find that they are less likely to use the Web Coach program as well. These findings are consistent with research that suggests that younger smokers are more ambivalent about quitting and less successful in their cessation efforts. We were somewhat surprised by the lower levels of engagement with this age group since they had proactively enrolled in the program on their own. The lower levels of engagement may suggest that younger smokers don’t realize the benefits of a program when they quit and/or that their expectations for the program were not met and thus they were less engaged. This requires further study. Japunitch et al [14] found no difference in use by gender, ethnicity, or education but found a difference by age. Strecher and colleagues [26] found that a tailored Web program was more effective for those who had a tobacco-related disease, who had nonsmoking children at home, and who frequently drank, but no other known correlates of outcomes (eg, gender, age, motivation to quit) were found to significantly predict program effectiveness.

### Limitations

There are some limitations that should be considered when interpreting the findings from this study. First, this is an evaluation of a real-world service and results are based on services people receive/use and not based on randomization to services. Thus, we are limited in our ability to make causal inferences regarding findings. Second, tobacco abstinence is based on self-report without biochemical verification. Since this a commercial service, biochemical verification of tobacco status is not a standard part of the services. It has not been deemed necessary in large studies of this kind and self-report has been considered adequate [27]. Third, the descriptive findings in this study are somewhat limited as there was a limited battery of measures included. Demographics were limited to age and gender. Lastly, it is notable that while many findings met statistical significance, measures of effect size tended to be small (eg, Cohen’s d = .00-.12); thus the extent to which these findings reflect clinically significant differences remains unclear.

### Conclusions

This study offers valuable information regarding the real-world use and effectiveness of a novel, integrated phone/Web program for tobacco cessation. Results indicate that more telephone counseling and greater use of the Web are associated with better quit outcomes. When provided access to both proactive phone counseling and Web-based services, smokers were more likely to utilize the phone over the Web. Yet those who chose to complement telephone counseling with Web services appeared to have experienced superior outcomes. Given the potential for increased efficiency and individual tailoring provided via Internet applications, and the strong evidence base for phone effectiveness, there is ample room for improvement in the development, promotion, and study of integrated approaches leveraging the phone and Web in order to further engage smokers with these modalities of treatment. Findings from this study add further support for health plans and employers to offer cessation services for their members and employees. Promoting both phone and Web-based components of an integrated program achieves the best results.
